# Study on association between shear wave elastography parameters and clinicopathological characteristics in breast cancer: A protocol for systematic review

**DOI:** 10.1097/MD.0000000000023082

**Published:** 2020-11-20

**Authors:** Hong-hong Xue, Yuan-yuan Wang

**Affiliations:** a Department of Ultrasound, Yanan University Affiliated Hospital; b Department of Breast Surgery, Yan’an People's Hospital, Yan’an, Shaanxi, China.

**Keywords:** association, breast cancer, clinicopathological characteristics, shear wave elastic parameters

## Abstract

**Background:**

This study aims to explore the association between shear wave elastography parameters (SWEPs) and clinicopathological characteristics (CPCs) in breast cancer (BC).

**Methods:**

The electronic databases of Cochrane Library, MEDLINE, EMBASE, Allied and Complementary Medicine Database, WANGFANG, VIP, and China National Knowledge Infrastructure will be used to search for studies dated from database inception to the present. No limitations of language and publication status will be applied in this study. Only case-controlled study and randomized controlled trials investigating the association between SWEP and CPC in BC will be included. Cochrane risk of bias will be used to assess study quality for each included study. RevMan 5.3 software will be utilized for statistical analysis.

**Results:**

This study will provide accurate data to appraise the association between SWEP and CPC in BC.

**Conclusion:**

This study will summarize the most recent evidence to improve our understanding of the association between SWEP and CPC in BC.

**OSF registration number:**

osf.io/vmkwu.

## 1 Introduction

Breast cancer (BC) is the most common type of cancer in female population worldwide.^[1–3]^ It is also the leading cause of cancer-related mortality and morbidity.^[4–6]^ In China, it is the most frequently diagnosed cancer in females over 30 years of age.^[7]^ It is reported that there were 1.38 million new cases of invasive BC identified globally in 2008. Patients with BC are often discovered by routine screening, and it often manifests as breast lump, change of breast shape and size, as well as nipple discharge.^[8–12]^ Several risk factors are responsible for BC, such as age, gender, personal history of BC, family history of BC, histologic, genetic, and reproductive risk factors, and exogenous hormone use.^[13–17]^

Studies reported that ultrasound shear wave elastography (SWE) can supply novel insights into the shear elastic modulus of soft tissues.^[18–23]^ It can be used to detect differences between healthy and pathological tissues.^[18–23]^ It is reported that ultrasound SWE has used for the detection of patients with BC.^[24–26]^ However, no study has performed the association between SWE parameters (SWEPs) and clinicopathological characteristics (CPCs) in BC. Thus, this study will explore this issue systematically and comprehensively.

## 2 Methods

### 2.1 Study registration

We have registered this study on OSF (osf.io/vmkwu), and we have reported it according to the Preferred Reporting Items for Systematic Reviews and Meta-Analysis Protocol statement guidelines.^[27]^

### 2.2 Ethics and dissemination

The ethical approval is waived in this study, because this study will use published patient data. This study is expected to be published in a peer-reviewed journal or present in a relevant conference.

### 2.3 Eligibility criteria

#### 2.3.1 Types of studies

All case-controlled study (CCS) or randomized controlled trials (RCTs) on exploring the association between SWEP and CPC in BC will be considered for inclusion in this study. All other studies, such as animal study, nonclinical study, and noncontrolled trial will be excluded.

#### 2.3.2 Types of interventions

Experimental group: we will include studies that involved the lump of breast in patients with BC.

Control group: we will include studies that involved adjacent tissue of attacked breast for examination.

#### 2.3.3 Types of participants

We will include patients who were diagnosed as BC, regardless their country, race, age, gender, and educational or economic status.

#### 2.3.4 Types of outcome measurements

Outcomes include lump hardness parameters (maximum elastic modulus, average, standard deviation), lesion size, pathological type, and axillary lymph node metastasis.

### 2.4 Information sources

A literature search will be conducted at Cochrane Library, MEDLINE, EMBASE, Allied and Complementary Medicine Database, Chinese Biomedical Literature Database, and China National Knowledge Infrastructure since their inception to the present. No language limitation is applied to all electronic databases. In addition, we will also search websites of clinical trial registry, conference proceedings, dissertations, and reference lists of included studies. A sample of search strategy for PUBMED is presented in Table 1. We will also adapt similar search strategy to other electronic databases.

**Table 1:** Search strategy of PubMed.

Number	Search terms
1	breast cancer
2	breast carcinoma
3	breast tumor
4	breast lump
5	Or 1–4
6	shear wave elastography
7	Ultrasound
8	shear elastic modulus
9	Operation
10	clinicopathological characteristics
11	Pathological
12	Parameters
13	Association
14	Or 6–13
15	Random
16	Randomly
17	controlled trial
18	clinical trial
19	Blind
20	Allocation
21	case-control
22	case-controlled
23	case-reference
24	Study
25	Trial
26	Or 15–25
27	5 and 14 and 26

### 2.5 Selection of studies

Two authors will independently scan the searched records using titles and abstracts. Any duplicated and irrelevant studies will be removed. Then, they will go through the full papers of the remaining studies in order to determine whether they finally meet all inclusion criteria. Any discrepancies between 2 authors will be solved by a third author through discussion. The process of study selection is summarized in a flowchart.

### 2.6 Data extraction and management

Two authors will independently extract the data according to the previously designed data extraction sheet that includes basic study details (such as author names, publication time, location, and so on), patient characteristics (such as age, sex, sample size, inclusion and exclusion criteria, and so on), study setting, study methods, index details, outcomes, results, findings, conflict of interest, and funding information. Any disagreements will be solved through discussion and will provide final judgment with the help of a third author if necessary.

### 2.7 Study quality assessment

Two authors will independently assess study quality for all included RCTs using Cochrane Risk of Bias Tool and all eligible CCS using Newcastle--Ottawa Scale. If any divergences occur between 2 authors, a third author will be invited to solve them by discussion.

### 2.8 Measures of treatment effect

We will utilize mean difference (MD) or standard MD and 95% confidence intervals (95% CIs) for continuous data, and will express risk ratio and 95% CIs for dichotomous data.

### 2.9 Heterogeneity identification

The heterogeneity among the study results will be assessed using *I*^2^ statistic. A value of *I*^2^ *≤* 50% exerts a low level of heterogeneity, whereas a value of *I*^2^ *>* 50% indicates an obvious level of heterogeneity.

### 2.10 Statistical analysis

#### 2.10.1 Data synthesis

In this study, statistical analysis will be carried out using RevMan 5.3 software. We will use a fixed-effects model if a low level of heterogeneity is found. We will consider to perform a meta-analysis if at least 2 studies on the same outcome with sufficient similarity are included. Otherwise, we will use a random-effects model if a substantial level of heterogeneity is identified. In addition, we will carry out subgroup analysis to explore any possible causes for such substantial heterogeneity. Whenever it is necessary, we will also plan to undertake a narrative summary description by presenting detailed written commentary on different study information, participant characteristics, and outcomes.

#### 2.10.2 Subgroup analysis

A subgroup analysis will be performed based on the different study information, patient characteristics, index details, and outcome measurements.

#### 2.10.3 Sensitivity analysis

We will carry out a sensitivity analysis in order to determine whether the outcome results are stability by removing studies with a high risk of bias.

#### 2.10.4 Reporting bias

If 10 or more eligible publications will be found, funnel plot and Egger regression test will be conducted to check any potential reporting bias.^[28,29]^

## 3 Discussion

BC is a very commonly diagnosed cancer in female population, which significant impacts their health-related quality of life. Patients with such disorder often have high mortality and morbidity rates. However, it is not easy to detect this condition at very early stage. Fortunately, ultrasound SWE has reported to detect differences between healthy and pathological tissues.^[18–23]^ Although several studies explored the association between SWEP and CPC in patients with BC, no systematic review has undertaken this topic. This study will systematically and comprehensively investigate the association between SWEP and CPC in BC. We expect that our study will provide evidence to improve our understanding of the association between SWEP and CPC in BC.

## Author contributions

**Conceptualization:** Hong-hong Xue, Yuan-yuan Wang.

**Data curation:** Hong-hong Xue, Yuan-yuan Wang.

**Formal analysis:** Hong-hong Xue, Yuan-yuan Wang.

**Funding acquisition:** Yuan-yuan Wang.

**Investigation:** Yuan-yuan Wang.

**Methodology:** Hong-hong Xue.

**Project administration:** Yuan-yuan Wang.

**Resources:** Hong-hong Xue.

**Software:** Hong-hong Xue.

**Supervision:** Yuan-yuan Wang.

**Validation:** Hong-hong Xue, Yuan-yuan Wang.

**Visualization:** Hong-hong Xue, Yuan-yuan Wang.

**Writing – original draft:** Hong-hong Xue, Yuan-yuan Wang.

**Writing – review & editing:** Hong-hong Xue, Yuan-yuan Wang.

## References

[1] Harbeck N Gnant M Breast cancer. Lancet 2017;389:1134–50. 27865536 10.1016/S0140-6736(16)31891-8

[2] Harbeck N Penault-Llorca F Cortes J Breast cancer. Nat Rev Dis Primers 2019;5:66. 31548545 10.1038/s41572-019-0111-2

[3] Pearce L Breast cancer. Nurs Stand 2016;30:15. 10.7748/ns.30.51.15.s16 27533387

[4] Grayson M Breast cancer. Nature 2012;485:S49. 22648496 10.1038/485S49a

[5] Shimozuma K Breast cancer. Gan To Kagaku Ryoho 2019;46:985–9. 31273161

[6] Gemignani ML Armstrong DK Breast cancer. Gynecol Oncol 2014;132:264–7. 24528543 10.1016/j.ygyno.2014.01.022

[7] Chen W Zheng R Baade PD Cancer statistics in China, 2015. CA Cancer J Clin 2016;66:115–32. 26808342 10.3322/caac.21338

[8] Barrett SV Breast cancer. J R Coll Physicians Edinb 2010;40:335–8. 21132144 10.4997/JRCPE.2010.418

[9] Jerusalem G Scheen AJ Breast cancer. Rev Med Liege 2011;66:225–8. 21826951

[10] Rasheed R Breast cancer. J Coll Physicians Surg Pak 2013;23:766–7. 24112273 10.2013/JCPSP.766767

[11] Morrow M Gradishar W Breast cancer. BMJ 2002;324:410–4. 11850376 10.1136/bmj.324.7334.410 PMC1122337

[12] Hindle W Breast cancer: introduction. Clin Obstet Gynecol 2002;45:738–45. 12370613 10.1097/00003081-200209000-00018

[13] Rojas K Stuckey A Breast cancer epidemiology and risk factors. Clin Obstet Gynecol 2016;59:651–72. 27681694 10.1097/GRF.0000000000000239

[14] Momenimovahed Z Salehiniya H Epidemiological characteristics of and risk factors for breast cancer in the world. Breast Cancer (Dove Med Press) 2019;11:151–64. 31040712 10.2147/BCTT.S176070 PMC6462164

[15] Doren A Vecchiola A Aguirre B Gynecological-endocrinological aspects in women carriers of BRCA1/2 gene mutations. Climacteric 2018;21:529–35. 30295091 10.1080/13697137.2018.1514006

[16] Stuckey A Breast cancer: epidemiology and risk factors. Clin Obstet Gynecol 2011;54:96–102. 21278508 10.1097/GRF.0b013e3182080056

[17] Broeders MJ Verbeek AL Breast cancer epidemiology and risk factors. Q J Nucl Med 1997;41:179–88. 9274126

[18] Chen YL Gao Y Chang C Ultrasound shear wave elastography of breast lesions: correlation of anisotropy with clinical and histopathological findings. Cancer Imaging 2018;18:11. 29622044 10.1186/s40644-018-0144-x PMC5887177

[19] Evans A Whelehan P Thompson A Identification of pathological complete response after neoadjuvant chemotherapy for breast cancer: comparison of greyscale ultrasound, shear wave elastography, and MRI. Clin Radiol 2018;73:910 e1-6. 10.1016/j.crad.2018.05.030 29980324

[20] Liu B Zheng Y Huang G Breast lesions: quantitative diagnosis using ultrasound shear wave elastography-A systematic review and meta--analysis. Ultrasound Med Biol 2016;42:835–47. 26778289 10.1016/j.ultrasmedbio.2015.10.024

[21] Gregory A Mehrmohammadi M Denis M Effect of calcifications on breast ultrasound shear wave elastography: an investigational study. PLoS One 2015;10:e0137898. 26368939 10.1371/journal.pone.0137898 PMC4569403

[22] Rzymski P Skórzewska A Skibińska-Zielińska M Factors influencing breast elasticity measured by the ultrasound Shear Wave elastography-preliminary results. Arch Med Sci 2011;7:127–33. 22291745 10.5114/aoms.2011.20617 PMC3258684

[23] Rzymski P Skórzewska A Opala T Changes in ultrasound shear wave elastography properties of normal breast during menstrual cycle. Clin Exp Obstet Gynecol 2011;38:137–42. 21793274

[24] Evans A Whelehan P Thompson A Prediction of pathological complete response to neoadjuvant chemotherapy for primary breast cancer comparing interim ultrasound, shear wave elastography and MRI. Ultraschall Med 2018;39:422–31. 28934812 10.1055/s-0043-111589

[25] Fujioka T Katsuta L Kubota K Classification of breast masses on ultrasound shear wave elastography using convolutional neural networks. Ultrason Imaging 2020;42:213–20. 32501152 10.1177/0161734620932609

[26] Vinnicombe SJ Whelehan P Thomson K What are the characteristics of breast cancers misclassified as benign by quantitative ultrasound shear wave elastography? Eur Radiol 2014;24:921–6. 24326756 10.1007/s00330-013-3079-4

[27] Moher D Shamseer L Clarke M Preferred reporting items for systematic review and meta-analysis protocols (PRISMA-P) 2015 statement. Syst Rev 2015;4:1. 25554246 10.1186/2046-4053-4-1 PMC4320440

[28] Sutton AJ Duval SJ Tweedie RL Empirical assessment of effect of publication bias on meta-analyses. BMJ 2000;320:1574–7. 10845965 10.1136/bmj.320.7249.1574 PMC27401

[29] Egger M Davey Smith G Schneider M Bias in meta-analysis detected by a simple, graphical test. BMJ 1997;315:629–34. 9310563 10.1136/bmj.315.7109.629 PMC2127453

